# Interactive effects of ivermectin and nitrogen levels on phytoplankton biomass and stress responses

**DOI:** 10.1007/s10646-026-03104-w

**Published:** 2026-06-10

**Authors:** Suleiman Dauda, Adriana Sturion Lorenzi, Maria Onma Onaji, Micheline Kézia Cordeiro-Araújo, Yusuf Okpanachi Ibrahim, Kala Musa Adamu, Idris Aisha Adam, Mariya Awwal Likoro, Mathias Ahii Chia

**Affiliations:** 1 Department of Botany, Ahmadu Bello University, Zaria, Nigeria; 2 Graduate Program in Microbial Biology, Institute of Biological Sciences, University of Brasília, UnB, Brasília, DF Brazil; 3 Department of Biology, Ahmadu Bello University, Zaria, Nigeria; 4 Postgraduate Programme in Botany, Department of Biological Sciences, State University of Feira de Santana, Feira de Santana, 44036-900 BA Brazil; 5 Department of Biology, Nigerian Army University, Biu, Nigeria; 6 Department of Biological Sciences, Abubakar Tafawa Balewa University, Bauchi, Nigeria; 7 Department of Ecology, Institute of Biological Sciences, University of Brasília, UnB, Brasília, DF Brazil; 8 Department of Ecology, Institute of Biosciences, University of Sao Paulo, Sao Paulo, Brazil

**Keywords:** Aquatic ecosystems, Microalgae, Cyanobacteria, Interaction effect, Pharmaceuticals risk assessment

## Abstract

Ivermectin (IV), a commonly used antiparasitic drug for livestock and humans, eventually finds its way into aquatic environments, primarily through fecal matter and livestock carcasses. The presence of IV in water bodies may pose a risk to non-target aquatic organisms, such as phytoplankton, whose primary habitat is the aquatic environment. The adverse effects of IV may be exacerbated by its interactions with organic matter and nutrients, such as nitrogen, in aquatic ecosystems. Here, the combined effects of IV with low and replete nitrogen on the biomass and oxidative stress responses of *Chlorella vulgaris*, *Selenastrum capricornutum*, and *Microcystis flos-aquae* were studied. Additionally, the microcystins production of *M. flos-aquae* under the treatment conditions was also measured. The results show that the interaction between IV and nitrogen levels mostly did not affect the biomass of *C. vulgaris*, *S. capricornutum*, and *M. flos-aquae*. IV interacted with replete nitrogen to increase oxidative stress parameters, and also stimulated the three species to produce higher cell densities. Similarly, the interaction between IV and low nitrogen stressed *M. flos-aquae* and increased its production of microcystins (MC). These findings provide valuable insights into the complex interactions between IV, nitrogen levels, and phytoplankton physiology, highlighting the adaptive mechanisms used by phytoplankton in response to these conditions.

## Introduction

The presence of pharmaceuticals in the environment raises concerns because these substances are biologically active and can affect the physiological state of a wide range of living organisms, primarily impacting the behavior, reproduction, and growth of the aquatic biota (Rico et al. 2021). Ivermectin (IV) is a semi-synthetic derivative of avermectin B1, a macrocyclic lactone produced by the actinomycete *Streptomyces avermitilis* (Garric et al. 2007; El-Saber Batiha et al. 2020) that is widely recognized for its effectiveness in treating parasitic infections caused by nematodes (helminths) and arthropods (ectoparasites) (Ashour 2019). IV interferes with glutamate-gated or γ-aminobutyric acid-related chloride channels in synapse membranes (Mesa et al. 2017; Patil et al. 2022). This broad-spectrum antiparasitic agent is used extensively in veterinary medicine, agriculture, and even in some cases, human health (Garric et al. 2007; Mesa et al. 2017). While beneficial for controlling parasites, concerns have emerged regarding its persistence and effects on non-target organisms in the environment (Brinke et al. 2010; Boonstra et al. 2011; Mesa et al. 2017, 2020). Non-target organisms like phytoplankton lack glutamate-gated or gamma-aminobutyric acid (GABA) chloride channels (Garric et al. 2007; Wolstenholme 2012; Qian et al. 2023), therefore synaptic channel modulation by IV does not apply to them. Some of the modes of action IV on phytoplankton include the inhibition of growth (Garric et al. 2007), and photosynthesis (Marques et al. 2023).

An important consideration is the environmental risk posed by IV residues entering aquatic ecosystems (Garric et al. 2007). Ivermectin is poorly metabolized in treated livestock and is largely excreted unmetabolized, with about 80–90% of the administered dose excreted via feces (Alvinerie et al. 1999), and less than 2% via urine (Garric et al. 2007; Chaccour et al. 2017). The predominant residue reported from livestock dung is the unchanged parent compound, although minor metabolites have been detected (Alvinerie et al. 1999; González Canga et al. 2008; Tipthara et al. 2021). Due to its lipophilic nature, IV can bind tightly to organic materials and sediment (Mesa et al. 2020). A half-live of 1–8 days in surface water has been repored for IV (Boonstra et al. 2011). Therefore, once in aquatic environments, IV can persist and accumulate in sediments, making it bioavailable to aquatic organisms for extended periods (Garric et al. 2007; Mesa et al. 2017). Even at low concentrations, IV has been shown to disrupt the behavior, reproduction, and growth of aquatic species (Garric et al. 2007; Boonstra et al. 2011). However, since typical environmental concentrations of IV range from 0.08 to 6.57 µg L^− 1^ (Oluwole et al. 2020), any potential effects at concentrations above 100 µg L^− 1^ are likely unrealistic in natural environments. Studies have shown that IV can adversely affect phytoplanktonic organisms in aquatic ecosystems. In microalgae, such as *Raphidocelis subcapitata* (formerly *Pseudokirchneriella subcapitata*), the EC_50_ was determined at concentrations of approximately 4,000 µg L^− 1^ (Garric et al. 2007), which means that at this concentration, IV was capable of inhibiting 50% of algae growth. Phytoplankton species, such as *Synechococcus elongatus* and *Pseudokirchneriella subcapitata* exposed to IV suffer oxidative stress, reduction in growth, and net photosynthetic rates that depend on the nature of exposure and concentration (Martin et al. 2021; Marques et al. 2023; Udebuani et al. 2023).

The persistence of IV in aquatic systems exacerbates its potential effects on phytoplankton over time (Garric et al. 2007). However, aquatic ecosystems undergo temporal changes in physical and chemical conditions. Nitrogen forms are principal among nutrient conditions, and they affect and limit the distribution of phytoplankton in aquatic ecosystems. Considering that nitrogen modulates the response of phytoplankton to xenobiotics (Chia et al. 2015) and acknowledging that sources of ivermectin, such as effluents from aquaculture and dairy industries, are typically nitrogen-rich, it is imperative to account for these interactions in ecotoxicological research. Nitrogen-enriched sources, including aquaculture, dairy discharge, and direct excretion, significantly contribute to the eutrophication of aquatic environments. Thus, it is crucial to investigate how these factors interact and influence the chemistry and response of phytoplankton to ivermectin. Phytoplankton populations demonstrate resilience and sensitivity to IV exposure through various mechanisms, but an initial approach involves understanding their susceptibility vis-à-vis the physiological stress they undergo. These interactions can modify the bioavailability and toxicity of ivermectin, thereby impacting the physiological and biochemical reactions of phytoplankton. As such, incorporating nutrient concentrations into ecotoxicity risk assessments of xenobiotics is essential for a comprehensive understanding of their ecological impacts and their effects on phytoplankton community dynamics (Chia et al. 2021). This approach is critical for predicting shifts in phytoplankton populations and understanding ecological succession.

We hypothesise that IV exposure will induce oxidative stress–mediated growth inhibition in freshwater phytoplankton, with nitrate levels modulating cellular defense responses and toxin production in *Microcystis flos-aquae*. Therefore, this study aimed to investigate the effects of ivermectin on the growth, pigment content, and antioxidant enzyme activities of *Selenastrum capricornutum*, *Chlorella vulgaris*, and *Microcystis flos-aquae*, as well as on microcystin production in *M. flos-aquae*, under varying nitrate concentrations. These species are model phytoplankton that are sensitive to xenobiotics and commonly alternate and co-occur in aquatic ecosystems (Connon et al. 2019). The factors controlling these processes are evolving due to the increasing levels of pollutants and their interactions with fluctuating nitrogen levels in aquatic ecosystems, highlighting the need to conduct these studies.

## Materials and methods

### Study organisms, culture conditions, and maintenance

The microalgal strains used for this study were: *C. vulgaris* UTEX 2714, purchased from freshwater microalgae culture collection from the University of Texas, USA, *M. flos-aquae* UTEX 2677 (a microcystin-producing cyanobacterial strain) and *S. capricornutum* Printz, both obtained from Gobler Laboratory of the State University of New York at Stony Brook, New York, USA. The microalgal strains were kept under controlled laboratory conditions in the Phycology Laboratory, Department of Botany, Ahmadu Bello University, Zaria, Nigeria, in a blue-green 11 (BG- 11) medium with a pH of 7.4 (Chia et al. 2018), a temperature of 23 ± 2 ^◦^C, and 16:8 h light: dark cycle (cool-white fluorescent lamps), at intensity of 30 µmol mˉ^2^ sˉ¹. BG-11 medium was sterilized 24 h before use by autoclaving (121 °C, 30 min). The microalgal strains were continually sub-cultured at their exponential growth phase to obtain accurate physiological data. Cultures were monitored daily to prevent clumping and ensure adequate distribution and absorption of nutrients.

### Experimental design

Ivermectin (C_95_H_146_O_28_), consisting of 90% 5-O-demethyl-22,23-dihydroavermectin A_1a_ (22,23-dihydroavermectin B_1a_) and 10% 5-O-demethyl-25-de(1-methylpropyl)-22,23-dihydro-25-(1-methylethyl) avermectin A_1a_ (22,23 dihydroavermectin B_1b_), manufactured by ACTIZA Pharmaceutical Pvt. Ltd. and obtained from a licensed local supplier (Rukayyah Pharmacy, Zaria, Nigeria). The drug was finely ground using a ceramic mortar and pestle and then dissolved in distilled water to prepare a stock solution, a standard approach for poorly soluble compounds in OECD algaltox assays (TG 201) (OECD, 2019). Two nitrogen conditions were studied: replete and low nitrogen conditions. The nitrogen was supplied as KNO_3_ at 1.1 × 10^− 3^ and 2.9 × 10^− 6^ M, representing replete and low nitrogen concentrations in aquatic ecosystems, respectively. Nitrogen was supplied as potassium nitrate (KNO₃); therefore, potassium concentrations covaried proportionally with nitrate levels, and no independent stoichiometric adjustment of potassium was performed (Larned 1998; Reynolds 2006). Prior to treatment exposure, exponentially growing cultures had an initial cell density of approximately 1 × 10⁵ cells mL⁻¹. Each algal species (*C. vulgaris*, *S. capricornutum*, and *M. flos-aquae*) was cultured in 100 mL of BG11 medium (Rippka et al. 1979) with the total nitrogen concentration adjusted to the low and replete concentrations shown above. For treatments with ivermectin (IV), the concentration of IV used was 30 µg L^− 1^. Although this value exceeds the typical environmental range reported for surface waters (0.08–6.57 µg L⁻¹; Oluwole et al. 2020), it remains substantially below reported acute toxicity thresholds. For instance, the 72-h EC50 for growth inhibition in the freshwater chlorophyte *R. subcapitata* has been reported at approximately 4,000 µg L⁻¹ (Garric et al. 2007). Therefore, the selected concentration represents a sublethal exposure level suitable for assessing physiological and biochemical responses rather than acute inhibition.This concentration of IV falls within the range (< 1.1–494.4 µg L^− 1^) reported in water bodies (Nessel et al. 1989; Mesa et al. 2020) as a result of the entry of feces from livestock treated with IV. The experimental cultures of the microalgal strains were exposed to replete nitrogen (RN; 1.1 × 10^− 3^ M nitrogen), replete nitrogen plus ivermectin (RN + IV; 1.1 × 10^− 3^ M nitrogen combined with 30 µg L^− 1^ IV), low nitrogen (LN; 2.9 × 10^− 6^ M nitrogen), and low nitrogen plus ivermectin (LN + IV; 2.9 × 10^− 6^ M nitrogen combined with 30 µg L^− 1^ IV). The cultures under low and replete nitrogen conditions without IV exposure served as nitrogen-specific controls for comparison with IV-treated groups. All treatments were performed in triplicate (*n* = 3), and measurements were conducted on each replicate culture. The cultures were then incubated for five days under the same controlled conditions as the stock cultures.

### Biomass and pigment content determination

Cell density was determined by collecting aliquots on days 1, 3, and 5. The cell densities of *S. capricornutum*,* C. vulgaris*, and *M. flos-aquae* were measured using direct microscopic counts with a light microscope and an improved Neubauer hemocytometer. Microscopic counts and absorbance (750 nm) readings were used to monitor the growth rate of *S. capricornutum*,* C. vulgaris* and *M. flos-aquae* (Liang et al. 2013).

Pigments were extracted from culture aliquots (10 mL) of *S. capricornutum*,* C. vulgaris*, and *M. flos-aquae* by centrifuging at 4000 rpm for 10 min, then the resulting biomass was suspended in 3 mL of 80% acetone (v/v) and refrigerated for 24 h in the dark. The absorbance of the extracted pigments was measured at 470, 653, and 666 nm using a UV-VIS spectrophotometer (Spectrumlab 752s). Chlorophyll *a*, *b*, and total chlorophyll concentrations were calculated using the equations given by Ritchie (2006).

### Oxidative stress markers levels measurement

Ten mL culture aliquots of *S. capricornutum*,* C. vulgaris*, and *M. flos-aquae* were centrifuged at 4000 rpm for 10 min. The resulting biomass was homogenized using a mortar and pestle in 3 mL of cold buffer (0.1 M phosphate buffer, pH 7.4), then centrifuged at 4000 rpm for 10 min, and the supernatant were utilized for the analyses.

Intracellular hydrogen peroxide was determined by mixing titanium chloride solution (133 µL, 0.1%) with 400 µL of sample extract, and the level was measured at 410 nm using a UV-VIS spectrophotometer (722 N, Delta Electronics, Nanjing, China) (Jana and Choudhuri 1982). The intracellular H_2_O_2_ concentration in pmol cell^− 1^ was estimated using an H_2_O_2_ calibration curve and an extinction coefficient of 0.281^− 1^ cm^− 1^.

For malondialdehyde measurement, 1 mL of the cell extract was mixed with 2 mL of TCA (10% trichloroacetic acid) and TBA (0.5% thiobarbituric acid) solution and heated in a water bath set at 90 °C for 15 min, then placed in an ice bath (Heath and Packer 1968). The absorbance was measured at 532 nm and 600 nm using a Spectrumlab 752s spectrophotometer. The MDA concentration was calculated using an extinction coefficient of 155 mM^− 1^ cm^− 1^.

### Antioxidant enzyme activity assays

Glutathione S-transferase (GST) activity was measured in *S. capricornutum*,* C. vulgaris* and *M. flos-aquae* cells using reduced glutathione (GSH) and 1-chloro-4-dinitrobenzene (CDNB) according to (Habig et al. 1974) method. The mixture contained 1 mL of 0.1 M phosphate buffer, 970 µL of 1 mM GSH, 10 µL of 1 mM CDNB, and 10 µL of enzyme extract. The absorbance of the mixture was measured at 430 nm using a UV–VIS spectrophotometer (722 N, Delta Electronics, Nanjing, China) for 3 min at 1 min intervals. A control mixture without enzyme extract accounted for non-specific substrate binding. GST activity was expressed as nKat per million cells.

Peroxidase (POD) activity was determined using Pyrogallol (0.05 M in 0.1 M phosphate buffer, pH 6.5) and H_2_O_2_ (1% in 0.1 M phosphate buffer, pH 6.5). 3.0 mL of the pyrogallol solution and 0.1 mL of extract were dispensed into a cuvette. Subsequently, 1% H_2_O_2_ (0.5 mL) was added to the mixture, followed by thorough mixing. Changes in absorbance at 300 nm were recorded at 30 s intervals for a duration of 3 min. The enzyme activity was represented as the change in absorbance units g^− 1^ fresh weight min^− 1^ (Reddy et al. 1985).

### Microcystin content of *M. flos-aquae* exposed to ivermectin under different nitrogen conditions.

At the end of the exposure period, total intracellular microcystins were extracted from *M. flos-aquae* cultures using 20 mL aliquots. Samples were centrifuged at 4,000 rpm for 10 min to obtain cell pellets, and the supernatant was discarded. The pellets were resuspended in 3 mL of 80% (v/v) methanol, vortex-mixed for 10 s, and subjected to three freeze–thaw cycles at − 20 °C to ensure complete cell lysis, following Otogo et al. (2021). After extraction, samples were centrifuged again (4,000 rpm, 10 min), and the methanolic supernatant was collected and diluted with distilled water to reduce the final methanol concentration to < 5% (v/v), thereby minimizing potential matrix interference with ELISA detection. Total microcystins were quantified using a Microcystins-ADDA ELISA 96-well plate kit (Eurofins Abraxis Inc., Warminster, PA, USA) according to the manufacturer’s instructions. The quantification of total intracellular microcystins was performed using a Bio-Rad iMark™ microplate absorbance reader (Bio-Rad Laboratories, Inc., Japan) at a wavelength of 450 nm. The concentrations were expressed in femtograms per cell (fg cell⁻¹).

### Statistical analysis

Two-way analysis of variance (ANOVA) and Repeated measures ANOVA (RMANOVA) was used to determine significant differences in the cell density, chlorophylls *a* and *b*, and total chlorophyll responses of each algal species and between the algal species to the treatments over time, respectively. One-way ANOVA was used to determine significant differences in oxidative stress responses and antioxidant enzyme activities of the algal species, and microcystin production of *M. flos-aquae* to the treatments. Two-way ANOVA was used to determine significant differences in the oxidative stress responses and antioxidant enzyme activities between the algal species. Prior to performing ANOVA and RMANOVA, Shapiro-Wilk test for normality, Levene’s test for homogeneity of variance, and Mauchly’s sphericity test were conducted. To determine which means were significantly different from others, Tukey’s HSD test was performed. A correlation-based principal component analysis (PCA) was used to assess the relationships between the biochemical stress-response parameters and the treatments for each algal species. All analyses were performed at a 5% significance level using R version 4.2.3.

## Results

### Growth and pigment content of phytoplankton exposed to different nitrate and ivermectin conditions

**Fig. 1:**
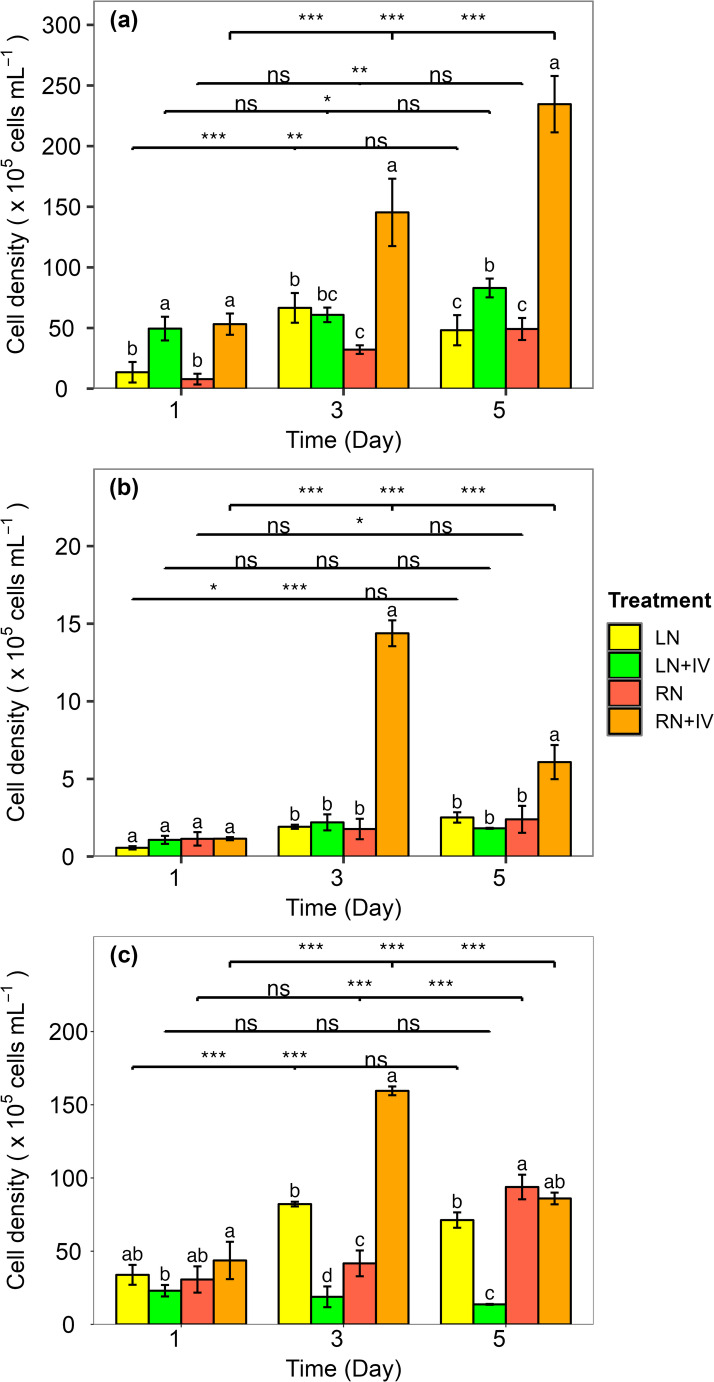
Cell density of *Chlorella vulgaris* (a), *Selenastrum capricornutum* (b), and *Microcystis flos-aquae* (c) treated with low nitrogen (LN), replete nitrogen (RN), a combination of low nitrogen with ivermectin (LN + IV) and replete nitrogen with ivermectin (RN + IV) as functions of time. Bars represent treatment mean ± sd (*n* = 3). Treatments with different letters within each sampling time are significantly different (Two-way ANOVA, *p* < 0.05). Temporal changes within each treatment are indicated by brackets with asterisks (* = *p* < 0.05; ** = *p* < 0.01; *** = *p* < 0.001; ns = *p* > 0.05)

Cell density of *C. vulgaris* varied significantly in response to treatment and exposure duration (Two-way ANOVA: F = 24.79, *p* < 0.001; Fig. 1a). On day 1, cultures exposed to LN + IV and RN + IV exhibited significantly greater cell densities compared with those subjected to LN and RN treatments alone. By days 3 and 5, the RN + IV treatment resulted in significantly higher cell densities than all other treatment conditions. Similarly, the cell density of *S. capricornutum* was significantly influenced by treatment and time (Two-way ANOVA: F = 94.68, *p* < 0.001; Fig. 1b). Significant increases in cell density were observed on days 3 and 5 under the RN + IV treatment relative to the other experimental conditions. For *M. flos-aquae*, cell density was likewise significantly affected by treatment over time (Two-way ANOVA: F = 69.24, *p* < 0.001; Fig. 1c). Exposure to RN + IV for 3 days led to a significant increase in cell density compared with the other treatments. Overall, species-specific responses to the treatments across time were significantly different, as indicated by repeated-measures ANOVA (F = 40.42, *p* < 0.001; Table 1). Notably, IV treatment consistently promoted algal growth across all three species, independent of nitrogen concentration.

**Table 1:** Repeated measures ANOVA summary table for growth and pigment content *Chlorella vulgaris*,* Selenastrum capricornutum*,* and Microcystis flos-aquae* exposed to ivermectin under different nitrate conditions. P-values with asterisks are significantly different at *p* < 0.05

Parameter	Source	df	Mean Sq	F value	Pr(> F)
Cell density	Error: ID				
	Treatment	3	1.96E + 14	277.54	2.01E-08*
	Residuals	8	7.05E + 11		
	Error: ID				
	Time	2	1.37E + 14	204.49	4.04E-12*
	Treatment: Time	6	2.64E + 13	39.49	1.03E-08*
	Residuals	16	6.68E + 11		
	Error: Within				
	Treatment	3	6.80E + 13	89.73	1.08E-19*
	Species	2	4.62E + 14	610.28	7.42E-35*
	Treatment: Time	6	1.83E + 13	24.18	5.87E-13*
	Treatment: Species	3	6.65E + 13	87.81	1.67E-19*
	Time: Species	4	4.32E + 13	57.02	1.21E-17*
	Treatment: Time: Species	6	3.06E + 13	40.42	3.93E-17*
	Residuals	48	7.58E + 11		
Chlorophyll *b*	Error: ID				
	Treatment	3	1.14	34.83	6.11E-05*
	Residuals	8	0.03		
	Error: ID				
	Time	2	7.62	186.09	8.33E-12*
	Treatment: Time	6	0.97	23.60	4.21E-07*
	Residuals	16	0.04		
	Error: Within				
	Treatment	3	2.04	48.66	1.36E-14*
	Species	2	2.35	55.95	2.86E-13*
	Treatment: Time	6	1.71	40.77	3.32E-17*
	Treatment: Species	3	1.29	30.84	2.93E-11*
	Time: Species	4	2.04	48.63	2.64E-16*
	Treatment: Time: Species	6	1.35	32.22	3.13E-15*
	Residuals	48	0.04		
Chlorophyll *a*	Error: ID				
	Treatment	3	0.31	29.01	1.19E-04*
	Residuals	8	0.01		
	Error: ID				
	Time	2	1.66	176.24	1.26E-11*
	Treatment: Time	6	0.13	13.96	1.45E-05*
	Residuals	16	0.01		
	Error: Within				
	Treatment	3	0.16	24.86	7.47E-10*
	Species	2	0.33	51.02	1.32E-12*
	Treatment: Time	6	0.21	32.21	3.15E-15*
	Treatment: Species	3	0.46	71.13	1.10E-17*
	Time: Species	4	0.41	63.82	1.29E-18*
	Treatment: Time: Species	6	0.26	41.36	2.50E-17*
	Residuals	48	0.01		
Total chlorophyll	Error: ID				
	Treatment	3	3.72	117.11	5.99E-07*
	Residuals	8	0.03		
	Error: ID				
	Time	2	11.20	292.94	2.49E-13*
	Treatment: Time	6	1.95	51.07	1.51E-09*
	Residuals	16	0.04		
	Error: Within				
	Treatment	3	2.68	62.45	1.35E-16*
	Species	2	1.86	43.34	1.77E-11*
	Treatment: Time	6	2.95	68.92	6.76E-22*
	Treatment: Species	3	3.54	82.68	5.62E-19*
	Time: Species	4	5.71	133.31	2.33E-25*
	Treatment: Time: Species	6	2.01	46.86	2.05E-18*
	Residuals	48	0.04		

**Fig. 2:**
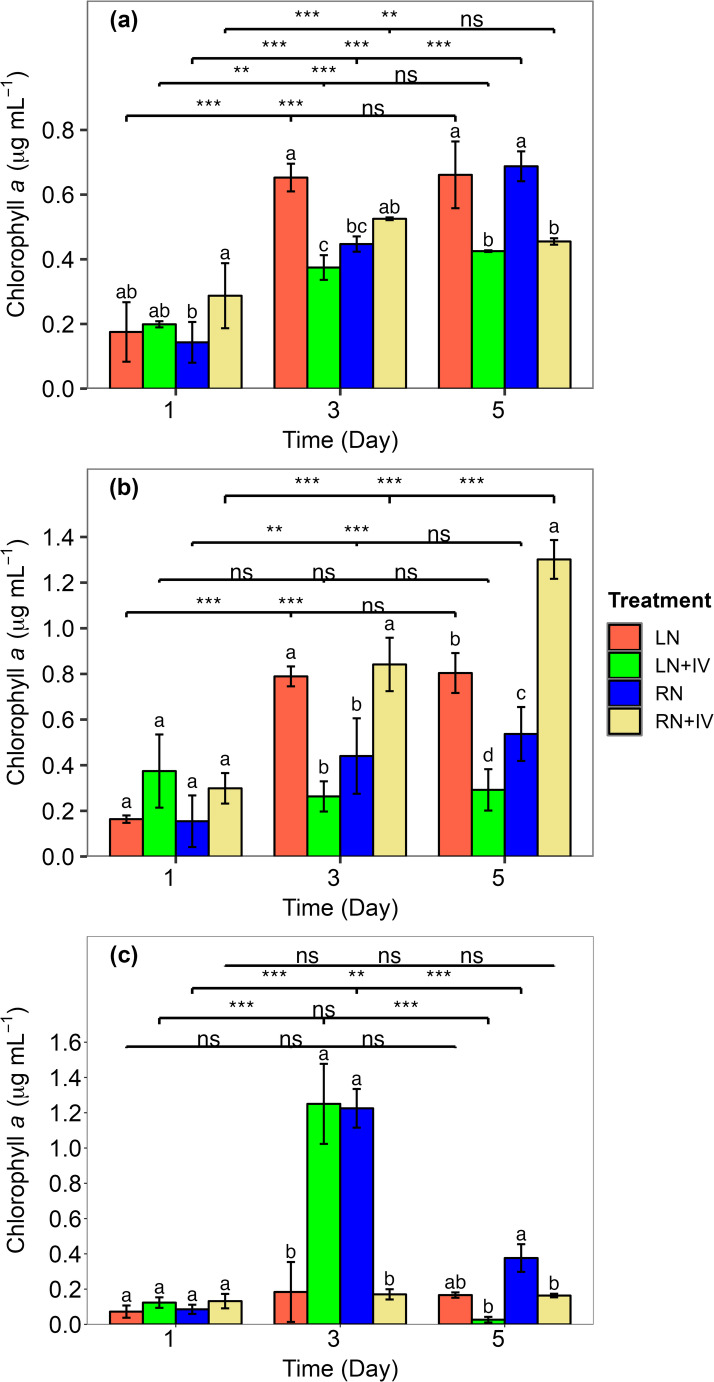
Chlorophyll *a* content of *Chlorella vulgaris* (a), *Selenastrum capricornutum* (b), and *Microcystis flos-aquae* (c), treated with low nitrogen (LN), a combination of low nitrogen with ivermectin (LN + IV), replete nitrogen (RN), and a combination of replete nitrogen with ivermectin (RN + IV), as functions of time. Bars represent treament mean ± sd (*n* = 3). Treatments with different letters within each sampling time are significantly different (Two-way ANOVA, *p* < 0.05). Temporal changes within each treatment over time are indicated by brackets with asterisks (* = *p* < 0.05; ** = *p* < 0.01; *** = *p* < 0.001; ns = *p* > 0.05)

The results of chlorophyll *a* content showed that the treatment conditions significantly affected *C. vulgaris* (Two-way ANOVA: F = 10.54, *p* < 0.001), *S. capricornutum* (Two-way ANOVA: F = 17.03, *p* < 0.001), and *M. flos-aquae* (Two-way ANOVA: F = 44.42, *p* < 0.001) over time (Fig. 2). The chlorophyll *a* content of *C. vulgaris* was significantly higher at LN and RN than LN + IV and RN + IV on day 5 (Fig. 2a). The chlorophyll *a* content of *S. capricornutum* significantly increased on day 5 at RN + IV (Fig. 2b). *M. flos-aquae* had a significant increase in its chlorophyll *a* under LN + IV and RN treatments on day 3 than LN and RN + IV treatments (Fig. 2c).

**Fig. 3:**
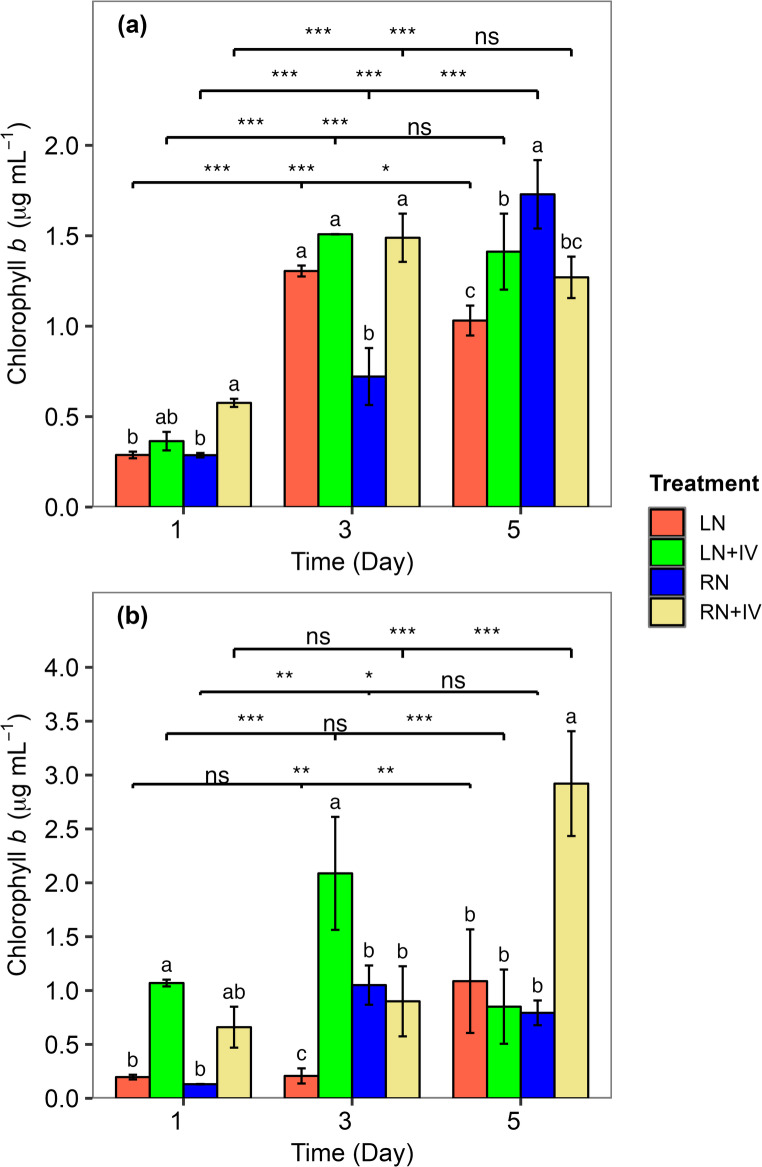
Chlorophyll *b* contents of *Chlorella vulgaris* (a), and *Selenastrum capricornutum* (b) treated with low nitrogen (LN), a combination of low nitrogen with ivermectin (LN + IV), replete nitrogen (RN), and a combination of replete nitrogen with ivermectin (RN + IV), as functions of time. Bars represent treament mean ± sd (*n* = 3). Treatments with different letters within each sampling time are significantly different (Two-way ANOVA, *p* < 0.05). Temporal changes within each treatment over time are indicated by brackets with asterisks (* = *p* < 0.05; ** = *p* < 0.01; *** = *p* < 0.001; ns = *p* > 0.05)

The chlorophyll *b* content of *C. vulgaris* (Two-way ANOVA: F = 23.78, *p* < 0.001) and *S. capricornutum* (Two-way ANOVA: F = 18.68, *p* < 0.001) was affected by treatments over time (Fig. 3). RN significantly reduced the chlorophyll *b* of *C. vulgaris* on day 3 (Fig. 3a), but on day 5 RN increased its chlorophyll *b* compared to LN and RN + IV. LN + IV on day 3 and RN + IV on day 5 significantly increased the chlorophyll *b* in *S. capricornutum* (Fig. 3b).

**Fig. 4:**
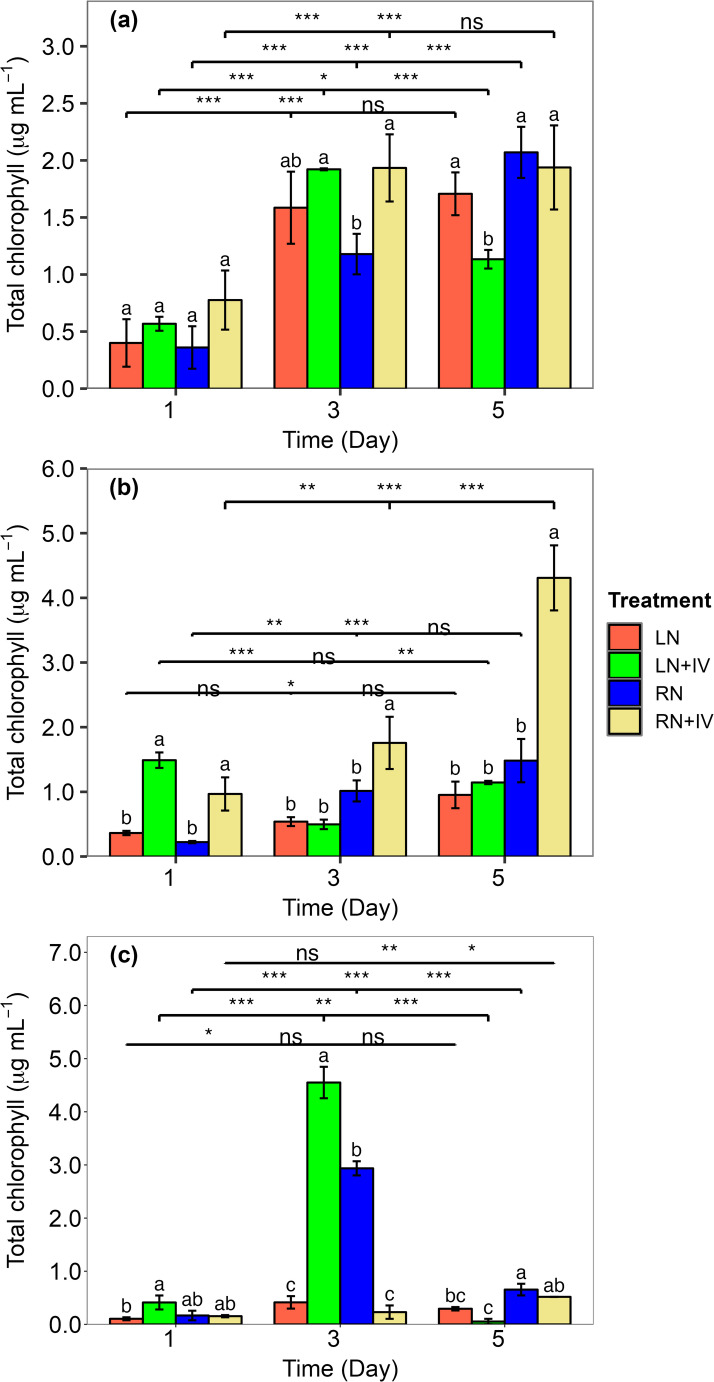
Total chlorophyll contents of *Chlorella vulgaris* (a), *Selenastrum capricornutum* (b), and *Microcystis flos-aquae* (c) treated with low nitrogen (LN), a combination of low nitrogen with ivermectin (LN + IV), replete nitrogen (RN), and a combination of replete nitrogen with ivermectin (RN + IV), as functions of time. Bars represent treament mean ± sd (*n* = 3). Treatments with different letters within each sampling time are significantly different (Two-way ANOVA, *p* < 0.05). Temporal changes within each treatment over time are indicated by brackets with asterisks (* = *p* < 0.05; ** = *p* < 0.01; *** = *p* < 0.001; ns = *p* > 0.05)

The total chlorophyll content of *C. vulgaris* (Two-way ANOVA: F = 7.54, *p* = 0.0013), *S. capricornutum* (Two-way ANOVA: F = 36.63, *p* < 0.001), and *M. flos-aquae* (Two-way ANOVA: F = 300.30, *p* < 0.001) (Fig. 4). The total chlorophyll content of *C. vulgaris* significantly reduced under LN + IV on day 5 (Fig. 4a). RN + IV significantly increased the total chlorophyll content of *S. capricornutum* on day 5 (Fig. 4b). The total chlorophyll content of *M. flos-aquae* was significantly increased by LN + IV, followed by RN on day 3 (Fig. 4c). On day 5, the LN + IV treatment had the lowest total chlorophyll content.

The influence of treatments on the cell density (RMANOVA: F = 40.42, *p* < 0.001), chlorophyll *a* (RMANOVA: F = 41.36, *p* < 0.001), b (RMANOVA: F = 32.22, *p* < 0.001), and total chlorophyll contents (RMANOVA: F = 46.86, *p* < 0.001) between the algal species were significantly different (Table 1).

### Oxidative stress response of phytoplankton species

**Fig. 5:**
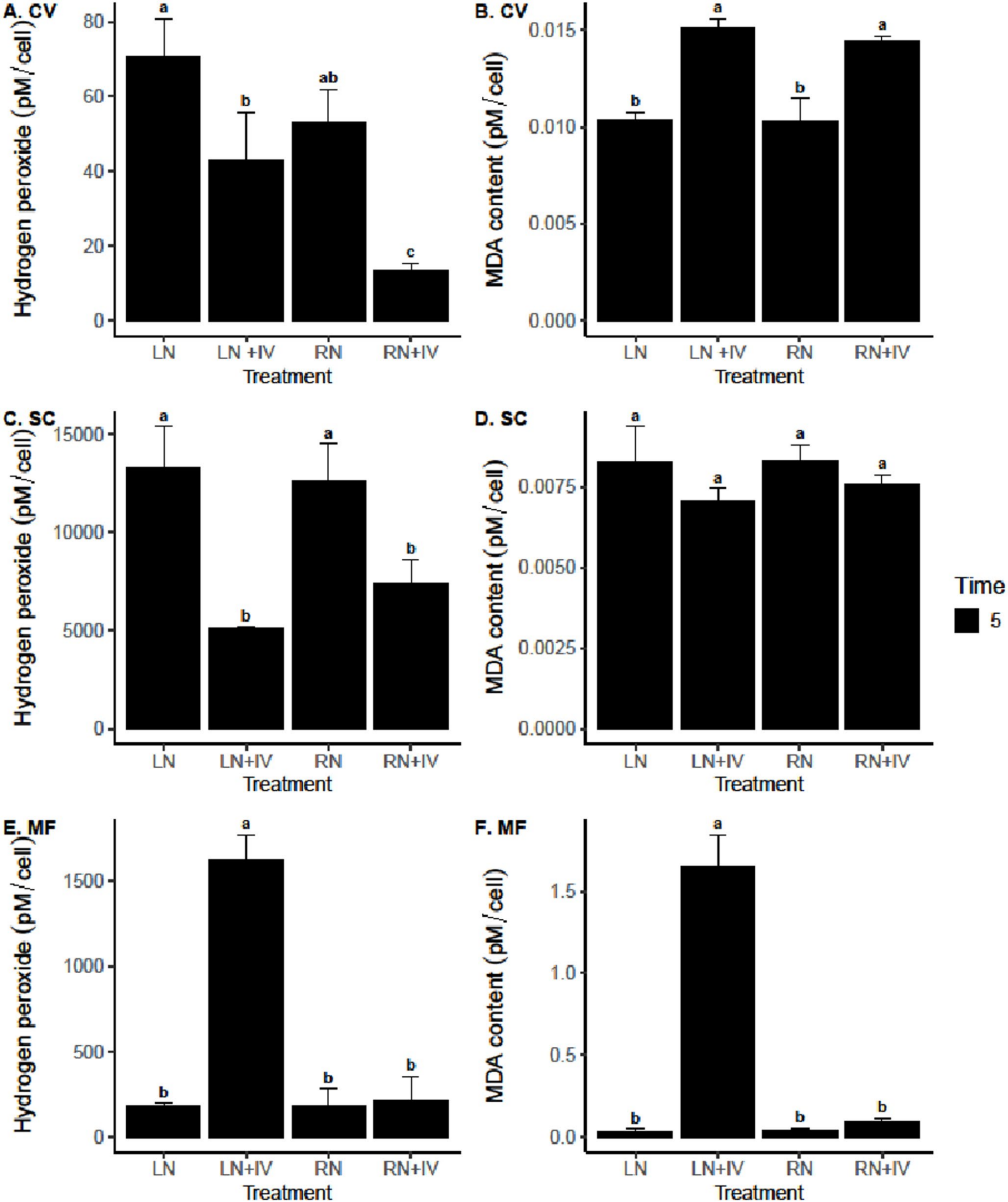
Hydrogen peroxide (a) and malondialdehyde contents (b) of *Chlorella vulgaris*, hydrogen peroxide (c) and malondialdehyde contents (d) of *Selenastrum capricornutum*, hydrogen peroxide (e) and malondialdehyde contents (f) of *Microcystis flos-aquae*, treated with low nitrogen (LN), a combination of low nitrogen with ivermectin (LN + IV), replete nitrogen (RN), and a combination of replete nitrogen with ivermectin (RN + IV), after 5 days of culturing. Bars represent mean ± sd (*n* = 3). Means with different alphabets are significantly different (One-way ANOVA, *p* < 0.05)

Nitrogen availability modulated antioxidant responses, with IV interacting with nitrogen to differentially alter ROS levels, lipid peroxidation, and antioxidant enzyme activities in a species-specific manner. The hydrogen peroxide (H_2_O_2_) content of *C. vulgaris* was significantly changed by the treatments (One-way ANOVA: F = 19.3, *p* = 0.0005) (Fig. 5a). Treatment of *C. vulgaris* with LN + IV and RN + IV significantly (*p* < 0.05) reduced its H_2_O_2_ content when compared to treatments with LN and RN only respectively. The treatment of *S. capricornutum* with LN + IV and RN + IV significantly (One-way ANOVA: F = 20.2, *p* = 0.0004) reduced its H_2_O_2_ content when compared to treatment with either LN or RN only (Fig. 5c). The H_2_O_2_ of *M. flos-aquae* was significantly (One-way ANOVA: F = 119.51, *p* < 0.001) increased only when it was treated with LN + IV in comparison to treatment with RN + IV, LN, and RN (Fig. 5e). The differences in the species response to the treatment conditions were significant (Two-way ANOVA: F = 23.43, *p* < 0.001; Table 2).

**Table 2:** Two-way ANOVA summary table for antioxidant response and total microcystins content of *Chlorella vulgaris*,* Selenastrum capricornutum*,* and Microcystis flos-aquae* exposed to ivermectin under different nitrate conditions. P-values with asterisks are significantly different at *p* < 0.05

Parameter	Source	df	Mean Sq	F value	Pr(> F)
GST	Treatment	3	4.12E-05	197.83	4.70e-17*
	Species	2	1.07E-04	514.10	1.98e-20*
	Treatment: Species	6	4.05E-05	194.45	3.94e-19*
	Residuals	24	2.08E-07		
ROS	Treatment	3	12196220.20	15.35	8.70e-06*
	Species	2	348630698.00	438.85	1.26e-19*
	Treatment: Species	6	18615843.90	23.43	6.29e-09*
	Residuals	24	794419.50		
MDA	Treatment	3	0.64	186.59	9.22e-17*
	Species	2	0.79	230.69	2.14e-16*
	Treatment: Species	6	0.63	185.70	6.76e-19*
	Residuals	24	0.00		
POD	Treatment	3	1.96E-05	75.478844	1.17e-09*
	Species	1	1.22E-06	4.708978	4.54e-02*
	Treatment: Species	3	3.84E-05	147.998968	7.04e-12*
	Residuals	16	2.60E-07		

Malondialdehyde (MDA) content was significantly (One-way ANOVA: F = 42.06, *p* < 0.001) increased in *C. vulgaris* when it was treated with LN + IV and RN + IV in comparison to treatment with LN or RN alone (Fig. 5b). Whereas the MDA content of *S. capricornutum* did not significantly (One-way ANOVA: F = 2.45, *p* = 0.14) change under all treatments (LN, LN + IV, RN, RN + IV) used in this study (Fig. 5d). The MDA of *M. flos-aquae* was significantly (One-way ANOVA: F = 186.01, *p* < 0.001) increased only when it was treated with LN + IV in comparison to treatment with LN, RN, and RN + IV (Fig. 5f). The differences in MDA content between the species to the treatment conditions were significant (Two-way ANOVA: F = 185.70, *p* < 0.001; Table 2).

### Antioxidant enzyme activities and microcystin content

**Fig. 6:**
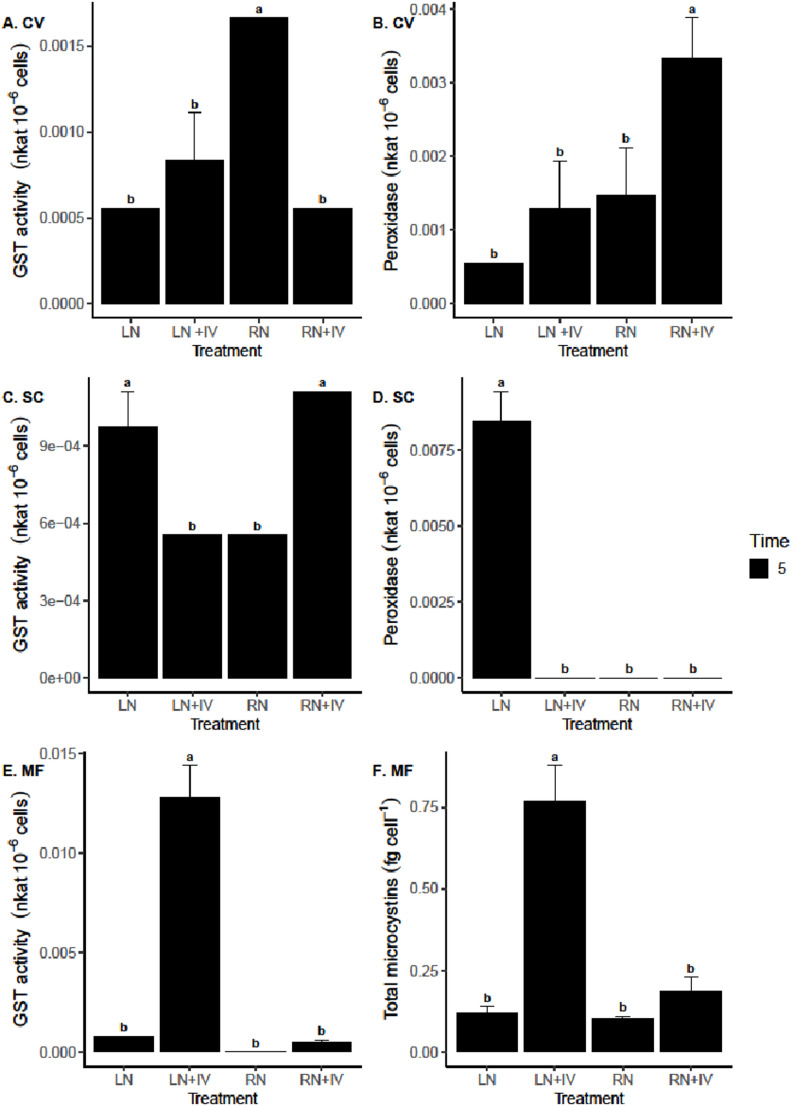
Glutathione S-transferase (a) and peroxidase activities (b) of *Chlorella vulgaris*, glutathione S-transferase (c) and peroxidase activities (d) of *Selenastrum capricornutum*, glutathione S-transferase (e) activity and microcystins content (f) of *Microcystis flos-aquae*, treated with low nitrogen (LN), a combination of low nitrogen with ivermectin (LN + IV), replete nitrogen (RN), and a combination of replete nitrogen with ivermectin (RN + IV), after 5 days of culturing. Bars represent mean ± sd (*n* = 3). Means with different alphabets are significantly different (One-way ANOVA, *p* < 0.05)

The glutathione S-transferase (GST) activity of *C. vulgaris* was significantly (One-way ANOVA: F = 43.0, *p* < 0.001) increased when it was treated with RN, in comparison to the LN, LN + IV, and RN + IV treatments (Fig. 6a). Whereas LN and RN + IV significantly (One-way ANOVA: F = 51.0, *p* < 0.001) increased the GST activity of *S. capricornutum* in comparison to LN + IV and RN (Fig. 6c). Only treatment with LN + IV significantly (One-way ANOVA: F = 201.64, *p* < 0.001) increased the GST activity of *M. flos-aquae* in comparison to LN, RN, and RN + IV (Fig. 6e). The peroxidase (POD) activity of *C. vulgaris* was significantly (One-way ANOVA: F = 14.79, *p* = 0.0013) increased when it was treated with RN + IV in comparison to LN, LN + IV, and RN (Fig. 6b). Only treatment with LN in comparison to LN + IV, RN, and RN + IV significantly (One-way ANOVA: F = 227.82, *p* < 0.001) increased the POD activity of *S. capricornutum* (Fig. 6d). In general, the changes in POD activity between *C. vulgaris* and *S. capricornutum* (Two-way ANOVA: F = 147.99, *p* < 0.001) and GST activity (Two-way ANOVA: F = 194.45, *p* < 0.001) between the investigated species were significantly different following exposure to the ivermectin under different nitrogen conditions (Table 2).

### Microcystin content variations of *M. aeruginosa* exposed to ivermectin under nitrogen conditions

In *M. flos-aquae*, the microcystin content (fg cell^− 1^) was significantly influenced by the LN + IV treatment (Fig. 6f), in which microcystin concentrations reached values up to 7 times higher than in the other treatments (One-way ANOVA: F = 85.26, *p* < 0.001).

### Relationships between the biochemical stress-response variables of *C. vulgaris*, *S. capricornutum*, and *M. aeruginosa* and ivermectin and nitrogen treatments

**Fig. 7:**
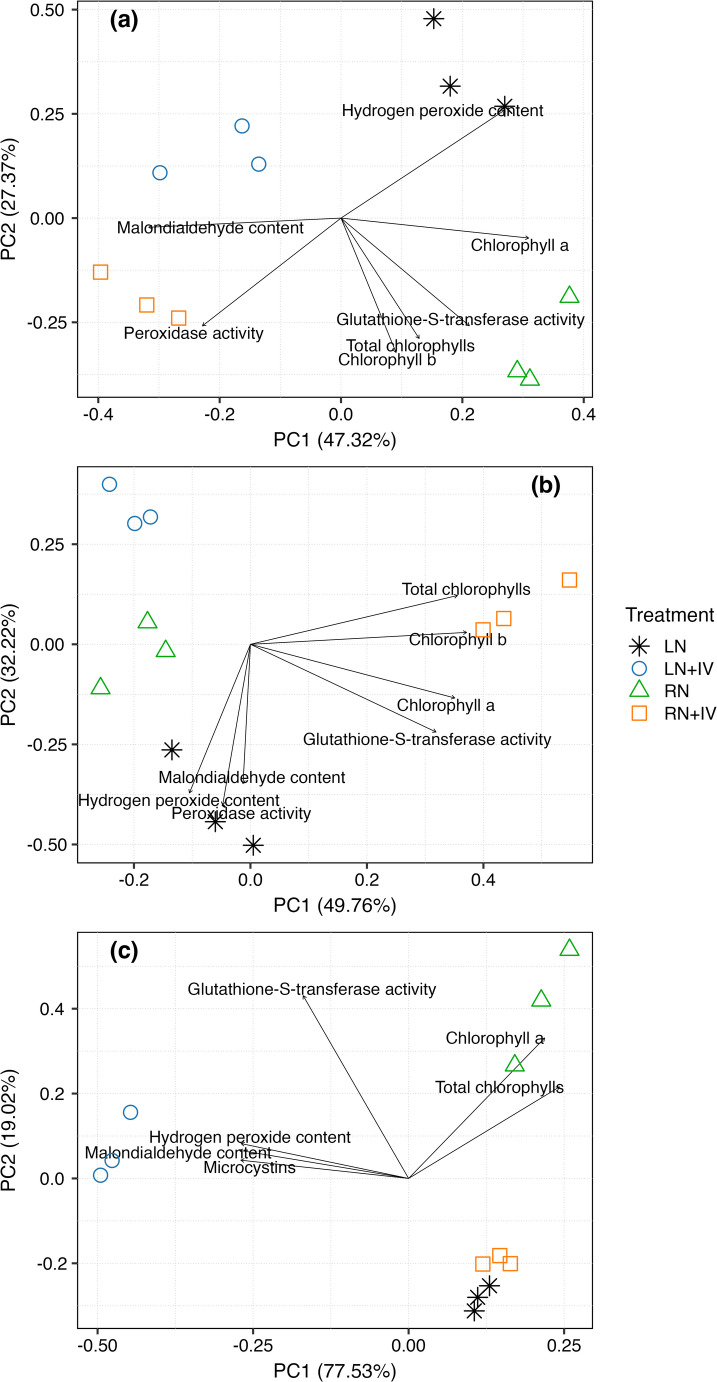
Principal components analysis (PCA) biplots of the relationships between the measured parameters of *Chlorella vulgaris* (a), *Selenastrum capricornutum* (b), and *Microcystis flos-aquae* (c), treated with low nitrogen (LN), a combination of low nitrogen with ivermectin (LN + IV), replete nitrogen (RN), and a combination of replete nitrogen with ivermectin (RN + IV), after 5 days of culturing

The principal components (PCs) 1 and 2 explained 74.69% of the total variation in response parameters of *C. vulgaris* to the treatments (LN, LN + IV, RN, and RN + IV) used in this study (Fig. 7a). Chlorophyll *a* content, ROS content, and GST activity of *C. vulgaris* were positively correlated with each other and negatively correlated with MDA content and POD activity. The total chlorophylls and chlorophyll *b* content of *C. vulgaris* were positively correlated. For *S. capricornutum*, PC1 and PC2 explained 81.98% of the total variation of the response parameters to the treatments (Fig. 7b). Chlorophylls *a*, *b*, and total chlorophylls contents of *S. capricornutum* were positively correlated with GST activity. Also, for *S. capricornutum*, the increase in ROS content, POD activity, and MDA content were positively correlated. The PCs 1 and 2 explained 96.55% of the total variation of the response parameters of *M. flos-aquae* to the treatments used in this study (Fig. 7c). Increase in MDA, microcystins, ROS contents, and GST activity were positively correlated with each other and negatively correlated with chlorophyll *a* and total chlorophylls content.

## Discussion

### Growth response and pigment content

Cell density drives the rate of cell proliferation in phytoplanktonic communities (Jin et al. 2024). Phytoplankton growth is influenced by nutrients (e.g., nitrogen and phosphorus), light availability, residence time, grazing, and others (Cira et al. 2016). Nitrate is the predominant form of nitrogen in water and the primary nutrient supporting phytoplankton growth, and this study showed that it interacted with ivermectin to influence the growth of the investigated phytoplankton. These findings point to the possibility that the presence of IV in water bodies may interact with nutrient availability. This is primarily because IV may directly affect phytoplankton (e.g., by inhibiting growth and photosynthesis activity) (Marques et al. 2023), or indirectly alter the ability of phytoplankton to utilize nutrients, including nitrate (Mesa et al. 2017, 2020).

Although pharmaceutical products alter the growth, metabolism, and photosynthesis of aquatic organisms (Wang and Xu 2016; Wang et al. 2017), we observed that the treatment RN + IV (replete nitrogen + ivermectin) significantly increased cell density in *C. vulgaris* and *S. capricornutum* after 3 and 5 days of exposure. Similar results were obtained for *M. flos-aquae* treated with RN + IV (replete nitrogen + ivermectin) and RN (replete nitrogen) after 3 and 5 days of exposure. This may suggest a significant effect on the growth rate of this phytoplankton species.

Our results challenge the hypothesis that ivermectin (IV) universally negatively affects phytoplankton growth, contrasting with findings from Garric et al. (2007). That study reported growth inhibition in the cyanobacterium *Synechococcus elongatus* and the green alga *C. vulgaris* when exposed to IV concentrations ranging from 38 to 4,000 µg L⁻¹. However, a critical distinction lies in the exposure levels: the concentrations employed by Garric et al. (2007) are substantially higher than those typically detected in aquatic environments, which range from 0.08 to 6.57 µg L⁻¹ (Oluwole et al. 2020). In contrast, the present study utilised a concentration of 30 µg L⁻¹, which, while at the upper bound of environmental relevance, is considerably closer to real-world scenarios than the supra-environmental levels used for standard ecotoxicological assays like EC50 determinations. This concentration was selected to: (1) represent a worst-case scenario at the upper bound of potential environmental contamination, (2) allow direct comparison with previous laboratory studies (such as Tišler and Kožuh 2006; which tested up to 20 mg L⁻¹), and (3) ensure detectable sublethal effects across multiple endpoints (growth, pigments, oxidative stress, and cyanotoxin production). This difference in concentration regimes likely accounts for the divergent observations, underscoring that toxicity outcomes at high, laboratory-specific doses may not accurately predict ecological effects under more realistic exposure conditions.

Adverse effects on algal growth are generally observed in studies testing higher concentrations of these drugs, concentrations that rarely reflect those found in natural waters. In a 72-hour growth inhibition test, it was observed that Abamectin, a substance related to IV, ranging from 1 to 20 mg L⁻¹, did not inhibit the growth of the chlorophyte *Scenedesmus subspicatus* at a concentration of 10 µg L⁻¹ (Tišler and Kožuh 2006). Consistent with this, our study found no growth inhibition at 30 µg L⁻¹ a concentration three times higher, further supporting that IV does not reduce growth of chlorophyte species at environmentally relevant levels.

The observed growth stimulation aligns with hormesis, a biphasic dose-response where low stressor concentrations stimulate while high concentrations inhibit biological processes (Schirrmacher 2021). Hormetic responses to pharmaceuticals are well-documented in phytoplankton, with antibiotics at sub-inhibitory levels enhancing nutrient uptake, photosynthetic efficiency, or stress acclimation pathways (Wang and Tao 2025). Here, ivermectin at 30 µg L⁻¹ likely triggered compensatory responses, with nitrogen-dependent effects most pronounced under replete conditions.

Species-specific sublethal effects emerged despite growth stimulation: ivermectin reduced chlorophyll a content in *C. vulgaris* and increased lipid peroxidation in both *C. vulgaris* and *M. flos-aquae*. This population-cellular dichotomy underscores the necessity of multi-endpoint ecotoxicological assessments, as physiological stress manifests independently of growth endpoints.

Chlorophyll a reduction in *C. vulgaris* under both nitrogen regimes suggest ivermectin-induced photosynthetic apparatus damage, despite concurrent RN + IV growth stimulation potentially reflecting altered resource allocation favouring cell division over photosynthetic maintenance. In *M. flos-aquae*, temporal dynamics (initial chlorophyll a increase followed by reduction) indicate early compensation preceding cumulative impairment (Liu et al. 2011). While chlorophyll *a* concentrations generally follow trends in cell density, the relationship is not strictly linear. Expressing chlorophyll *a* as a cellular quota could provide further insight into the effects of the treatments on pigment allocation. Future studies incorporating pigment cell quotas would help differentiate growth-related responses from changes in cellular pigment content.

Pharmaceutical ingredients such as antibiotics can inhibit photosynthesis in phytoplankton species through damage to thylakoid membranes, electron transport inhibition, reduction in the synthesis of many essential proteins in the PSII reaction center and the cytochrome complex, and reduction in photosynthetic pigment content (Liu et al. 2011; Chia et al. 2021). In the present study, the chlorophyll content in the green microalgal and cyanobacterial species under ivermectin and nitrogen treatments presented distinct responses. While in *C. vulgaris*, the ivermectin treatments reduced the chlorophyll *a* content, in *S. capricornutum*, the nitrogen conditions had an evident impact together with ivermectin causing an increase in chlorophyll *a* under replete nitrogen with ivermectin and reduction under low nitrogen with ivermectin treatments. However, in *M. flos-aquae*, low nitrogen with ivermectin and replete nitrogen treatments increased the pigment in the middle of the experiments. At the end of the experiments, the ivermectin treatments (LN + IV and RN + IV) reduced this pigment content. These findings demonstrate very important physiological and adaptation processes that the extent of growth inhibition, photosynthesis, and reduction in photosynthetic pigment content depend on microalgal and cyanobacterial species or strain, and pharmaceutical exposure conditions, and that these effects are vary.

Ivermectin up to 3.2 µg L^− 1^ did not affect the growth rate of the cyanobacterium *Synechococcus elongatus*, but negatively affected the growth of the green microalga *C. vulgaris* (Marques et al. 2023). The authors suggested that different responses between the species may be due to differences in the absorption and elimination of the pharmaceutical via degradation or enzymatic activity and in the efflux pump proteins activity. The effects of three antibiotics (erythromycin, ciprofloxacin, and sulfamethoxazole) on the photosynthesis process of *S*. *capricornutum* indicated a significant inhibition on the primary photochemistry, electron transport, photophosphorylation, and carbon assimilation (Liu et al. 2011). Considering experiments with concentrations of 25 to 100 µg L^− 1^ of the antibiotics florfenicol and thiamphenicol, the chlorophyll *a* content of the *Microcystis flos-aquae*, in a dose-concentration relationship, was significantly reduced, which was attributed as a result of the disintegration of thylakoid membranes (Wang et al. 2017). However, under 1 µg L^− 1^ treatment, the chlorophyll content was significantly higher than the control group at the end of experiments (day 7), reinforcing that the effects are directly related to the applied doses and the type of pharmaceutical. These chlorophyll content results are similar to those found in our study with ivermectin treatments of experiments with the cyanobacterium *M*. *flos-aquae.* As described above in our results, the *M*. *flos-aquae* chlorophyll *a* content seems to be nitrogen-dependent in addition to the ivermectin. Fundamental macronutrients such as nitrogen and phosphorus are crucial regulators in controlling microalgal response to antimicrobial drugs (Khan et al. 2018; Le et al. 2023). A study showed that spiramycin, a macrolide antibiotic and antiparasitic with the function of protein synthesis inhibitor, promoted growth inhibition in *Microcystis aeruginosa* (Liu et al. 2015). The authors found that at high levels of nitrogen (5 to 50 mg L^− 1^), the increase in spiramycin concentration led to a decrease in the cyanobacterium’s growth, while nitrogen-deficient conditions alone stimulated its growth.

Furthermore, among cyanobacteria, there are more resistant species and others less resistant to variations in light intensity, temperature, nutrient availability, and hydrological regimes (Moore et al. 2005; de Tezanos Pinto et al. 2016). For example, atmospheric nitrogen-fixing, such as species that have specialized cells (heterocytes), cyanobacteria possess physiological characteristics that provide adaptive advantages under nitrogen-limited conditions, which can influence community composition in environments subjected to fluctuations (de Tezanos Pinto et al. 2016).

### The ivermectin mode of action in other organisms

The main mechanism of action of IV is common across many invertebrate taxa. In nematodes and arthropods, the pharmaceutical binds to GluCls (glutamate-gated chloride channel). This binding causes irreversible channel opening, sustained chloride influx, neuronal hyperpolarization, and subsequent paralysis or death (Martin et al. 2021). GluCls are absent in vertebrates; however, IV can interact with other cysteine loop receptors, specifically GABA (γ-aminobutyric acid) gated chloride channels. P-glycoprotein (P-gp) limits penetration into the central nervous system in mammals. However, in neonates or in those deficient in P-gp, IV administration results in brain accumulation, causing neurotoxicity and mortality, as exemplified in mice (Menez et al. 2012). IV brain accumulation was also observed in sea bream (*Sparus aurata*) following intraperitoneal administration, due to active efflux across the blood-brain barrier (Katharios et al. 2004).

### Comparison of species resilience: oxidative stress and antioxidant enzymes response

In this study, we employed oxidative stress markers, intracellular hydrogen peroxide as an indicator of reactive oxygen species production, and MDA as an indicator of lipid peroxidation. The results from this study show differences in the oxidative responses of the green microalgae and cyanobacterium to the combination of nitrogen levels and ivermectin. The combination of nitrogen levels with ivermectin decreased the amount of H_2_O_2_ in *C. vulgaris* and *S. capricornutum*, the green microalgae tested in this study. The recorded decrease in H_2_O_2_ in *C. vulgaris* resulted from increased POD activity, an antioxidant defense mechanism by the cell to protect itself by converting H_2_O_2_ to other radicals.

The presence of ivermectin among biomolecules and exogenous pro-oxidants results in a loss of antioxidant defense in living organisms (Selaković et al. 2023). Combining nitrogen levels with ivermectin (LN + IV and RN + IV) in *C. vulgaris* and only LN + IV in *M. flos-aquae* caused higher lipid peroxidation in their cells. This implies that nitrogen and ivermectin acted synergistically to cause the oxidation of storage and membrane lipids in the cells of these organisms. However, the cells of *S. capricornutum* did not show any oxidation of its lipids by the treatments used in this study. While lipid peroxidation, likely due to the cellular and storage lipids damage, increased with an increase in ROS (H_2_O_2_) in *C. vulgaris* and *M. flos-aquae*, the lipid peroxidation and increase in oxidative stress in *S. capricornutum* were not related to the increase in ROS (H_2_O_2_). This is because the MDA content of this species was not altered by the treatments investigated in this species. Also, other types of ROS, like superoxide anions or hydroxyl ions, could have caused the increase in GST and POD activities in *S. capricornutum*. However, the phase II enzyme GST was actively involved in detoxification and transformation of ivermectin under limited nitrogen condition, and this process involved the use of reduced glutathione as a conjugate to biotransform xenobiotics in cyanobacteria (Potęga 2022; Cassier-Chauvat et al. 2023). It is also important to consider that GST also sequesters excessive ROS content that could be associated with limited nitrate conditions in cyanobacteria, and this may also be responsible for the increase in activity found under LN + IV exposure condition.

### Changes in microcystins concentrations and implications for aquatic ecosystem health

The highest microcystin concentrations in *M. flos-aquae*, in our study, were found in treatments under ivermectin and low nitrogen, confirming the relevance of the interaction of ivermectin and the investigated nitrogen conditions. These important findings demonstrate that cyanotoxin production can be enhanced by the interaction between pharmaceutical contaminants and nutrient limitation. While eutrophication remains a primary factor of cyanobacterial blooms and microcystin release, the present results show that high toxin concentrations can also occur under low nitrogen conditions when combined with additional stressors such as ivermectin. In agreement with our findings, the combination of nitrogen-deficient conditions and spiramycin (macrolide antibiotic and antiparasitic) stimulated the growth of *M. aeruginosa* and suggested a possible effect on microcystin production (Liu et al. 2015). In another study with *M. aeruginosa* under an antibiotic experiment (oxytetracycline, 1 to 10 mg L^− 1^), MC-LR equivalents (fg cell^− 1^) were oxytetracycline increase dose-dependent (Ye et al. 2017). These findings are particularly important because cyanobacteria have an important role in ecosystem balance; however, conditions that promote the recurrent formation of cyanobacterial blooms are beginning to be recognized to go beyond eutrophication by increased nitrogen and phosphorus concentrations and changing global climate (Metcalf and Codd 2020). Emerging evidence, supported by the present study, corroborates with other findings pointing to modulation by pollutants such as herbicides and pharmaceuticals (Brêda-Alves et al. 2021; Chia et al. 2021). The impact of pharmaceuticals depends on the type, concentration, nature of exposure, and cyanobacterial strain or species exposed, and this is a major cause of concern as the number, complexity, and concentrations of pharmaceutical products and their degradation products continue to increase in aquatic ecosystems (Ye et al. 2017; Chia et al. 2021). Thus, the presence of pharmaceuticals in aquatic environments represents a real and worrying issue because these compounds are capable of increasing the levels of cyanotoxins and their ecotoxicological risks. This concern is driven by available literature showing that pharmaceuticals stimulate the formation of cyanobacterial blooms (Liu et al. 2017; Le et al. 2023). The effects of these contaminants on the aquatic community are evidenced by several studies, and increases in cyanotoxin production, as discussed here, is a further concern since their effects can be disastrous, including for human health.

## Conclusion

Phytoplankton resilience to IV is affected by nitrate availability and by interactions between the two conditions. Our findings point to a potential disruptions of ecosystem dynamics that can be driven by alterations in nutrient fluxes, which could amplify phytoplankton sensitivity to IV. Ecosystem disturbances such as nitrate availability or fluctuations in nitrogen concentrations may impair recovery mechanisms and lead to prolonged ecological impacts. Our results challenge the hypothesis that IV negatively affects the phytoplankton populations, because we found growth stimulation in some cases when IV was present regardless of nitrogen condition, albeit dependent on the time or duration of exposure. Our findings on IV and nitrate availability highlight the intricate relationships among chemical stressors, nutrient dynamics, and ecosystem responses. Addressing these complexities is essential for sustainable environmental management, ensuring the resilience of phytoplankton populations and, ultimately, communities and protecting aquatic ecosystem health in the face of ongoing environmental challenges.

## Data Availability

No datasets were generated or analysed during the current study.
